# LC-ESI/LTQOrbitrap/MS Metabolomic Analysis of Fennel Waste (*Foeniculum vulgare* Mill.) as a Byproduct Rich in Bioactive Compounds

**DOI:** 10.3390/foods10081893

**Published:** 2021-08-15

**Authors:** Maria Assunta Crescenzi, Gilda D’Urso, Sonia Piacente, Paola Montoro

**Affiliations:** Dipartimento di Farmacia, Università Degli Studi di Salerno, via Giovanni Paolo II n. 132, I-84084 Fisciano, SA, Italy; mcrescenzi@unisa.it (M.A.C.); gidurso@unisa.it (G.D.); piacente@unisa.it (S.P.)

**Keywords:** waste, bioactive compounds, nutraceutical products, mass spectrometry, metabolomic, biological properties

## Abstract

Food industries produce a high amount of waste every year. These wastes represent a source of bioactive compounds to be used to produce cosmetic and nutraceutical products. In this study, the possibility to retrain food waste as a potential source of bioactive metabolites is evaluated. In particular, metabolite profiles of different parts (bulb, leaves, stems and little stems) of fennel waste were investigated by liquid chromatography coupled with mass spectrometry (LC-ESI/LTQ Orbitrap MS). To discriminate the different plant parts, a Multivariate Data Analysis approach was developed. Metabolomic analysis allowed the identification of different metabolites mainly belonging to hydroxycinnamic acid derivatives, flavonoid glycosides, flavonoid aglycons, phenolic acids, iridoid derivatives and lignans. The identification of compounds was based on retention times, accurate mass measurements, MS/MS data, exploration on specific metabolites database and comparison with data reported in the literature for *F. vulgare*. Moreover, the presence of different oxylipins was relieved; these metabolites for the first time were identified in fennel. Most of the metabolites identified in *F. vulgare* possess anti-inflammatory, antioxidant and/or immunomodulatory properties. Considering that polyphenols are described to possess antioxidant activity, spectrophotometric tests were performed to evaluate the antioxidant activity of each part of the fennel.

## 1. Introduction

*Foeniculum vulgare* (Apiaceae/Umbellifearae), also known as fennel, is a perennial aromatic plant, originally from Asia Minor and Mediterranean regions [1]. At present, it is widespread to all temperate zones, prefers arid ground and does not tolerate cold or humid climates. Fennel presents straight stems with an intense green-blue color that can grow to heights of up to 2.5 m. The stems are finely divided into leaves composed of many filiform segments. The sheath leaves form the white bulb wrapping around the stems at the base. The flowers are a terminal part of the plant, yellow umbels 5–15 cm wide. Aromatic and carminative fruits reach a length of 4–10 mm and are surrounded by dried seeds [1,2,3].

In the last years, vegetable waste has received a lot of attention as an ingredient intended to produce cosmetics and nutraceuticals [4,5,6,7]. The waste of *F. vulgare* is a good source of polyphenols thus making it a great by-product with anti-inflammatory, antioxidant, immunomodulatory and apoptotic properties [8,9,10]. Several species belonging to Umbelliferae have been shown to influence immunity, playing a central role in a variety of diseases [11,12,13]. Thus, a diet rich in polyphenol compounds like flavonoids (such as quercetin and rutin) and coumarins can help to prevent diseases such as cancer, cardiovascular and neurodegenerative pathologies [14,15,16]. The bioactivity of polyphenols such as chlorogenic acid, ferulic acid and caffeic acid, is correlated with the increase in the activity of human lymphocyte proliferation and high-level production of INF-γ, enhancing the efficacy of host defense against intracellular pathogenesis and cancer [14,17,18].

Inflammation is normally related to oxidative stress in chronic disease, in which there is an increase of free radicals, such as superoxide, hydroxyl and peroxyl radicals. The imbalance of these chemical species may trigger atherosclerosis, cataract and inflammation [19]. Phenolic compounds and flavonoids have shown antioxidant activity, through several mechanisms, such as free radical inhibition, peroxide decomposition or oxygen scavenging [20,21]. Traditional medicine uses fennel to treat a wide range of health disorders linked to the digestive, reproductive, respiratory and endocrine systems due to its many pharmacological activities [1]. 

Fennel’s industrial processes create large quantities of by-products that may be rich in bioactive compounds. Thus, this work evaluates the possibility of recovering these wastes to use them in the formulation of nutraceuticals or cosmetics. 

Metabolomics is a large-scale analytical approach and lends itself well to the identification of specialized metabolites of plant species [22,23]. In this work, extracts obtained from different parts of the plant *F. vulgare* were analyzed through a Metabolite Profiling approach by using liquid chromatography coupled with mass spectrometry equipped with an electrospray source and orbitrap analyzer (LC-ESI/LTQOrbitrap/MS), which allowed the identification of several metabolites mainly belonging to hydroxycinnamic acid derivatives, flavonoid glycosides, flavonoid aglycons, phenolic acids, iridoid derivatives and lignans. LTQ Orbitrap can perform experiments in high-resolution mass spectrometry (HRMS) measuring accurately the molecular weight of the compounds revealed. Liquid chromatography coupled with tandem mass spectrometry (LC-MS^n^) was able to separate, fragment and then characterize most of the plant metabolites from a vegetable source [24]. LC-ESI-FT-MS is typically applied and coupled with multivariate statistics and pathway analysis to obtain the relevant information [22]. 

So, the resulting data were processed with a multivariate statistical analysis approach, using Principal Component Analysis (PCA) and Partial Least Squares Discriminant Analysis (PLS-DA), projection methods. This allowed the classification of different parts of the fennel in relation to the metabolites characterizing them as markers. 

Finally, spectrophotometric experiments have shown the waste of *F. vulgare* to be a good resource for producing functional ingredients for cosmetics and nutraceuticals. In fact, the antioxidant properties of extracts were evaluated by DPPH and TEAC assays, giving promising results. 

## 2. Materials and Methods

### 2.1. Raw Materials

By-products of *Foeniculum vulgare* were provided by Paolillo (Eboli, Salerno, Italy), a company specialized in the production and marketing of fennel. The waste was recovered from the processing of fennel of the variety Tiziano cultivated in the locality of Campomarino in Puglia and harvested in December 2019. Parts of the fennel supplied were the white leaves forming the bulb, the superficial leaves and both the large stems and the smaller stems. Based on this, the samples were divided into the following four groups: FVBU—*Foeniculum vulgare* Bulb, FVST*—Foeniculum vulgare* Stem, FVLS—*Foeniculum vulgare* Little Stem, FVLE—*Foeniculum vulgare* Leaf. 

### 2.2. Reagents and Solvents

Ethanol and water used for the extractions as well as the acetonitrile and water used for sep-paks were purchased from VWR (Milan, Italy). Acetonitrile (ACN), formic acid, water and methanol of LC-MS grade were supplied by Merk (Merk KGaA, Darmstadt, Germany). ABTS (2,2′-azino-bis(3-ethylbenzothiazoline-6-sulfonic acid)), Trolox (6-hydroxy-2,5,7,8-tetramethylchroman-2-carboxylic acid), DPPH (2,2-Diphenyl-1-picrylhydrazyl), NaOH (sodium hydroxide), NaNO_2_ (sodium nitrite), AlCl_3_ (aluminum chloride), K_2_S_2_O_8_ (potassium persulfate)_,_ PBS (Phosphate Buffered Saline), rutin and quercetin were purchased from Sigma-Aldrich (Milan, Italy).

### 2.3. Sample Preparation

Once the different parts of the fennel were divided, they were stored at −80 °C and then freeze-dried. The freeze-dried plant materials were extracted with two types of extraction: sonication with a solution of ethanol/water (80:20) and water decoction. 

As regards the sonication of FVBU and FVST samples 1 g of dried drugs were extracted with 20 mL of ethanol/water (80:20) for 15 min in the ultrasonic bath. On the other hand, 40 mL for 1 g of the matrix was required to extract the FVLS and FVLE samples. The extraction was repeated three times, filtering the extracts with filter paper. Decoction was carried out following EP pharmacopeia guidelines using 5 g of each sample in 100 mL of water. Only for the decoctions, a further purification step was required by using C18 cartridges (Phenomenex, Aschaffenburg, Germany). The cartridges were activated with 15 mL of 100% CH_3_CN, then conditioned with 15 mL of 10% CH_3_CN. One gram of sample was loaded over the column. For elution, a gradient starting from 10% CH_3_CN/H_2_O followed by 30% CH_3_CN/H_2_O and ending with 100% CH_3_CN has been applied. The extracts were dried using a stream of nitrogen and then, for the LC-MS analysis, a solution of water/methanol (50:50) with a final concentration of 1 mg/mL of the extract was prepared for each sample. 

### 2.4. LC-ESI/LTQOrbitrap/MS

Analysis and identification of specialized metabolites occurring in decoctions and ethanolic extracts were carried out by developing a method using an HPLC coupled with a hybrid mass spectrometer, which combines the linear trap quadrupole (LTQ) and Orbitrap mass analyzer. The experiments were executed with a Thermo scientific liquid chromatography system, equipped with a quaternary Accela 600 pump and an Accela autosampler, combined with a Linear Trap-Orbitrap hybrid mass spectrometer (LTQ-Orbitrap XL, Thermo Fisher Scientific, Bremen, Germany) equipped with an electrospray ionization (ESI) source. A Phenomenex Luna C18 5μm (150 × 2.00 mm) column (Phenomenex Aschaffenburg, Germany) was used to perform the separation. The mobile phases employed were water + 0.1% formic acid (A) and acetonitrile + 0.1% formic acid (B). An increasing linear-gradient (*v*/*v*) at a flow rate of 0.200 mL/min of solvent B was used: 0–10 min, from 5 to 15%; 10–30 min, from 15 to 35%; 30–40, from 35 to 80% and then back to 5% for 10 min [9]. The mass spectrometer operated in negative ion mode and 10 μL of each sample was used for injection. ESI source parameters were the following: capillary voltage −48 V; tube lens voltage −176.47 V; ion source temperature 280 °C; sheath and auxiliary gas flow (N_2_), 15 and 5; sweep gas 0; capillary voltage 3.5 kV. The full range *m*/*z* adapted to the acquisition of MS spectra was 180–1400. For the fragmentation study, a data-dependent scan was set up through which the precursor ions corresponding to the most intensive peaks were fragmented in the LC–MS analysis with a collision energy of 30%. Xcalibur software version 2.2 was used for instrument control, data acquisition and data analysis.

### 2.5. Multivariate Data Analysis

To better classify the samples and evaluate the different expressions of specialized metabolites in the four sample classes of *F. vulgare* (FVBU, FVST, FVLS, FVLE), both the LC-ESI/LTQ-Orbitrap/MS chromatograms of the ethanolic extracts and those of the decoctions were subjected to chemometric analyses such as Principal Component Analysis (PCA) and Partial Least Square Discriminant Analysis (PLS-DA). Each chromatogram was processed using open-source software for mass-spectrometry data processing called MZmine (http://mzmine.sourceforge.net/) accessed on 20 July 2020. This software allowed compensating retention time and removing noise from LC-MS profiles setting the noise level to 1.0 × 10^4^, so the data points below this value were not considered in the multivariate analysis. The dataset was exported and processed by SIMCAP+ software 12.0 (Umetrix AB, Umea Sweden) for the PCA, a projection method that allows samples to be grouped based on similarity in the composition of metabolites. The data were scaled to Unit Variance before multivariate data analysis. After PCA, PLS-DA was also carried out to identify the different metabolites between the various groups of the samples [25]. In this analysis, numeric values were assigned to the four classes of samples (FVBU, −2; FVST, −1; FVLS, +1; FVLE, +2) to use the regression algorithm necessary to classify the information on the different types of samples.

### 2.6. DPPH• Radical Scavenging Activity

The antiradical activity of the various parts of *F. vulgare* was evaluated using the stables 1,1-diphenyl-2-picrylhydrazyl radical (DPPH•) and the procedure previously described [26].

A methanolic solution of DPPH• with a concentration of 0.025 g/L was prepared. An aliquot (37.5 µL) of the methanolic solution containing different amounts of each extract (0.625–1.25–2.5 and 5 mg/mL) was added to 1.5 mL of DPPH• solution previously prepared. The control tubes were prepared by adding equal volume (37.5 µL) of the vehicle alone in 1.5 mL of DPPH• solution. The incubation time of the reaction was 10 min. After this time, the absorbance at 517 nm was measured on a UV-visible spectrophotometer (Spectrophotometer Multiskan Go, Thermo Scientific). All experiments were carried out in triplicate.

The extracts with antioxidant activity reduce the radical DPPH• in the compound DPPH-H, causing a reduction in absorbance. So, the antiradical activity of the extracts was assessed as a decrease of the absorbance at 517 nm, more precisely expressed as the percentage of the radical inhibition of DPPH according to Equation (1):

(1) $$\%\;\mathrm{Inhibition}\;\mathrm{DPPH}•=\frac{A_{0}-A_{e}}{A_{0}}\times 100$$

*A*_0_ is the average of the absorbances of the control in triplicate and *A*_e_ is the average of the absorbances of each concentration of the various extracts in triplicate. 

### 2.7. Trolox Equivalent Antioxidant Capacity (TEAC) Assay

The antioxidant capacity of the extracts was determined by the Trolox Equivalent Antioxidant Capacity (TEAC) assay as previously reported [27]. The extracts were diluted with methanol/water producing solutions with the following concentrations: 250, 500, 750, 1000 μg/mL. The assay was set up in the 96-well plates, combining 15 µL of each sample with 150 µL of ABTS. The absorbance was measured at 734 nm. All experiments were carried out in triplicate.

The percent decrease of absorbance was determined for each concentration relative to a blank absorbance (methanol/water) and was plotted as a function of the concentration of compound or standard, 6-hydroxy-2,5,7,8-tetramethylchroman-2-carboxylic acid (Trolox).

The antioxidant activities are expressed as a TEAC value, that is, the concentration of a standard Trolox solution with the same antioxidant capacity as 1 mg/mL of the tested extract; quercetin 3-*O*-glucoside was used as a reference compound.

### 2.8. Total Flavonoid Assay

The total flavonoid content was measured using the Allumine Chloride colorimetric assay using rutin as a standard following the procedure previously described with slight modification [28]. Then, 1 mL of each sample with a concentration of 1 mg/mL and 4 mL of water was added in a 10-milliliter volumetric flask. After this, 0.3 mL of 5% NaNO_2_ was added to the flask and then, 5 min 0.3 mL of 10% AlCl_3_ was also added. After 6 min, 2 mL 1 M NaOH was added and the end-water was added to the solution to reach the volume of 10 mL. The solutions were mixed well, and the absorbance was measured against the blank control at 510 nm on a UV-visible spectrophotometer. 

The content of flavonoids in the various extracts was expressed in rutin equivalents (RE) according to Equation (2):

(2) $$\mathrm{Flavonoid}\;\mathrm{amount}=\frac{A\;*\;m_{0}\;*\;10}{A_{0}\;*\;m}$$

Flavonoid amount was expressed in mg/g plant extracts in RE; in the equation, *A* is the average of the absorbance of extract in triplicate, *A*_0_ is the average of the absorbance of standard rutin solution in triplicate, *m* is the weight of the plant extract analyzed in g and *m*_0_ is the weight of the rutin in the solution in g. 

## 3. Results

### 3.1. Identification of Metabolites in F. vulgare Extracts by LC-ESI/LTQOrbitrap/MS and LC-ESI-LTQOrbitrap/MS/MS Analysis

**Figure 1:**
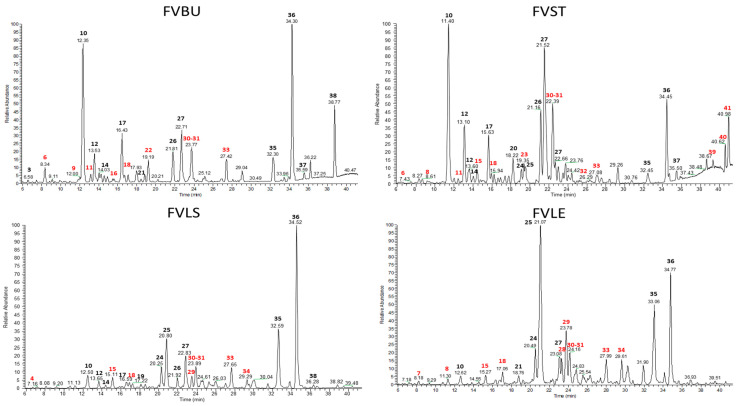
Negative ion mode profiles of *F. vulgare* waste ethanolic extracts obtained by LC-ESI/LTQ/Orbitrap MS: FVBU (*Foeniculum vulgare* Bulb), FVST (*Foeniculum vulgare* Stem), FVLE (*Foeniculum vulgare* Leaf) and FVLS (*Foeniculum vulgare* Little Stem). The compounds marked in red were identified only in the ethanolic extracts and not in the decoctions.

**Figure 2:**
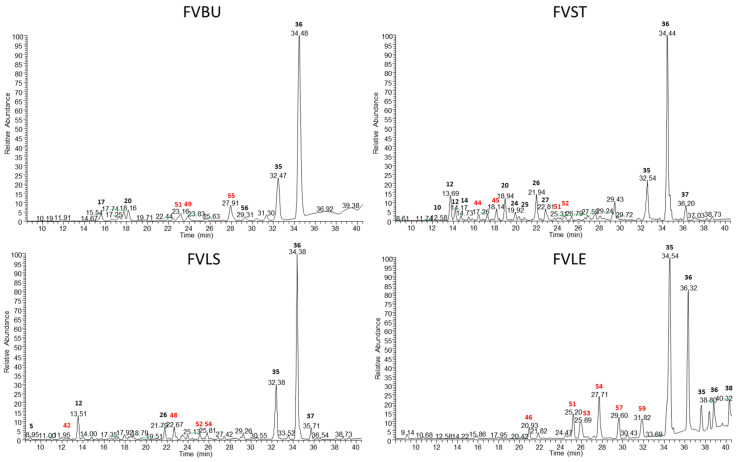
Negative ion mode profiles of *F. vulgare* waste decoctions obtained by LC-ESI/LTQ/Orbitrap MS: FVBU (*Foeniculum vulgare* Bulb), FVST (*Foeniculum vulgare* Stem), FVLE (*Foeniculum vulgare* Leaf) and FVLS (*Foeniculum vulgare* Little Stem). The compounds marked in red were identified only in the decoctions and not in the ethanolic extracts.

Metabolite profiles of hydroalcholic and decoction extracts obtained from waste of *F. vulgare*, were analyzed by LC-ESI-LTQ-MS/MS and investigated by Xcalibur Software (Figure 1 and Figure 2). The accurate mass measurement (ppm ≤ 5) and fragmentation experiments (MS/MS), associated with research in specific databases on spectral data for natural substances as KNApSAcK (www.knapsackfamily.com) accessed on 20 July 2020 and in the literature for the species *F. vulgare* allowed the putative identification of secondary metabolites mainly belonging to flavonoid glycosides (22–25,28–29), phenolic glycosides (4,51), coumarins (11 and 19), phenolic acids (1-5-6-10-16-17-27-31-32), iridoid derivates (2-7-12-13-18), lignans (14-43-44-50), oxylipins (35-36-54) and lipids (9, 39, 40 and 41) (Table 1 and Table 2). 

Among the metabolites previously identified in *F. vulgare*, flavonoid glycosides were found in fennel waste like flavonoid-*O*-rhamnoglucoside (22), for which the presence of the rhamnoglucoside unit was ascertained by checking neutral loss at 308 a.m.u.

Also, compounds 23 e 24 showed a fragmentation pattern corresponding to flavonoid glycosides. These compounds showed, in the LC-ESI/LTQOrbitrap/MS spectrum, product ions at *m*/*z* 285 and 301 peculiars of kaempferol and quercetin, respectively, originated by a sugar loss.

Other flavonoid glycosides, already present in the fennel literature, found in the waste of *F. vulgare* are flavonoid-*O*-glucuronides such as quercetin-*O*-glucuronide (25), luteolin-*O*-glucuronide (28), isorhamnetin-*O*-glucuronide (29), which were identified by a neutral loss scan of 176 a.m.u. corresponding to the uronic acid unit.

Peaks of *m*/*z* 353.0872, 367.1026, 337.0920 revealed the presence of chlorogenic acid (10), feruloyl quinic acid derivate (17) and coumaroylquinic acid (16), whereas a peak of *m*/*z* 515.1182 showed dicaffeoylquinic acid (27), phenolic acids previously identified in *F. vulgare* [9]. 

Other metabolites identified for the first time in fennel, previously reported in the Umbelliferae family, were the polyphenolic compound 4 (myrciaphenone A), the phenolic acid 5 (4-glucopyranosyloxy-3-methoxy benzeneacetic acid), the coumarin derivate 11 (fraxin) and the two lignans 14 (glehlinoside C) and 44 (secoisolariciresinol *O*-hesoside).

**Table 1:** Metabolites identified in *F. vulgare* waste ethanolic extracts by LC-ESI/LTQOrbitrap/MS and LC-ESI/LTQOrbitrap/MS/MS analysis. If the compound is present in several parts of the fennel, the RT is referred to as the profiles of FVBU, except for the compounds with * which are referred to as the profiles of FVLE.

N°	RT	[M − H]^−^	Molecular Formula	Δppm	MS/MS	Identity	FVBU	FVST	FVLS	FVLE	References
1	5.37	299.0764	C_13_H_15_O_8_	0.8	137.0248	4-hydroxybenzoic acid 4-*O*-glucoside	√			√	[9]
2	5.99	389.1078	C_16_H_21_O_11_	0.0	181.0505	eustomoside	√				[29]
3	6.58	218.1029	C_9_H_16_O_5_N	2.8	88.0408	pantothenic acid	√	√			[30]
4	7.16	329.0874	C_14_H_17_O_9_	2.1	167.0354	myrciaphenone A			√		[31]
5	8.27	343.1037	C_15_H_19_O_9_	3.8	181.0511/328.0800	4-glucopyranosyloxy-3-methoxy benzeneacetic acid		√			[32]
6	8.34	299.0765	C_13_H_15_O_8_	1.2	137.0247/179.0349/239.0556	4-hydroxybenzoic acid 4-*O*-glucoside	√	√			[9]
7	9.09	373.1142	C_16_H_21_O_10_	3.4	196.0381/211.0614	swertiamarin		√			[33]
8	11.30	293.1238	C_12_H_21_O_8_	2.5	131.0718	4-carboxy-1-methylbutyl glucopyranoside				√	[34]
9	11.79	387.1648	C_18_H_27_O_9_	−0.4	357.1552	tuberonic acid glucoside	√				[35]
10	12.36	353.0872	C_16_H_17_O_9_	1.4	191.0560	neochlorogenic acid	√	√	√	√	[9]
11	13.08	369.0815	C_16_H_17_O_10_	−0.4	207.0299	fraxin	√	√			[36]
12	13.53	583.2016	C_27_H_35_O_14_	−0.9	327.1230/375.1439/537.1968	lucidumoside C	√	√	√		[37]
13	13.78	355.1025	C_16_H_19_O_9_	0.3	175.0394/193.0508/217.0508	gentiopicrin	√				[38]
14	14.21	551.1754	C_26_H_31_O_13_	−0.9	341.1011/389.1225	glehlinoside C	√	√	√		[39]
15	15.27 *	351.1293	C_14_H_23_O_10_	2.1	249.0615/333.1194	unknown		√	√	√	
16	15.52	337.0920	C_16_H_17_O_8_	0.6	191.0560	3-*O*-p-coumaroylquinic acid	√				[9]
17	16.43	367.1026	C_17_H_19_O_9_	0.7	191.0558	3-*O*-feruloylquinic acid	√	√	√		[9]
18	16.70	371.0973	C_16_H_19_O_10_	0.0	249.0610	deacetylasperuloside	√	√	√	√	[34]
19	17.22	191.0349	C_10_H_7_O_4_	5.5	176.0117	6-*O*-methylesculetin			√		[40]
20	18.22	565.1933	C_27_H_33_O_13_	3.1	327.1238/339.1239	unknown		√			
21	18.76 *	425.1440	C_20_H_25_O_10_	−0.4	263.0919	1-benzopyran-6-propanoic acid, 7-glucopyranosyloxy-3,4-dihydro-2,2-dimethyl-4-oxo-	√			√	[41]
22	19.19	609.1444	C_27_H_29_O_16_	−1.0	301.0345	quercitin-3-*O*-rutinoside	√				[9]
23	19.35	447.0936	C_21_H_19_O_11_	3.2	285.0407	kaemferol 3-*O*-glucopyranoside		√			[9]
24	20.25 *	463.0872	C_21_H_19_O_12_	0.3	301.0356	quercitin 3-*O*-glucoside		√	√	√	[9]
25	20.80 *	477.0666	C_21_H_17_O_13_	0.4	301.0357	quercitin *O*-glucuronide		√	√	√	[9]
26	21.81	605.1862	C_29_H_33_O_14_	−0.5	339.1225	unknown	√	√	√		
27	22.71	515.1182	C_25_H_23_O_12_	−0.5	353.0871	1,3-dicaffeoylquinic acid	√	√	√	√	[9]
28	23.27	461.0721	C_21_H_17_O_12_	1.5	285.0406	luteolin-7-*O*-glucuronide				√	[9]
29	23.78 *	491.0822	C_22_H_19_O_13_	0.4	315.0519	isorhamnetin 3-*O*-β-D-glucuronide			√	√	[9]
30	24.16 *	233.0662	C_9_H_13_O_7_	2.5	173.0452	unknown	√	√	√	√	
31	24.16 *	601.1186	C_28_H_25_O_15_	−0.3	395.0974/439.0869/515.1185/557.1294	malonyl-1,4-*O*-dicaffeoylquinic acid	√	√	√	√	[42]
32	25.33	529.1359	C_26_H_25_O_12_	3.4	353.0883/367.1037	caffeoylferuloylquinic acid		√			[43]
33	27.42	483.1858	C_23_H_31_O_11_	−0.6	441.1753	unknown	√	√	√	√	
34	29.61 *	763.2443	C_36_H_43_O_18_	−0.2	337.1292	unknown			√	√	
35	33.06 *	327.2172	C_18_H_31_O_5_	1.8	211.1337/229.1439/291.1958	trihydroxy-octadecadienoic acid II	√	√	√	√	[44]
36	34.30	329.2325	C_18_H_33_O_5_	0.9	211.1339/229.1441/293.2115/311.2221	trihydroxy-octadecaenoic acid II	√	√	√	√	[45]
37	35.59	363.1804	C_20_H_27_O_6_	0.4	301.1800/345.1699	oridonin	√	√	√		[46]
38	38.77	293.1750	C_17_H_25_O_4_	1.1	221.1544/236.1048	gingerol	√				[47]
39	39.25	675.3610	C_33_H_55_O_14_	3.5	397.1356/415.1463	gingerglycolipid A		√			[48]
40	40.62	564.3317 [(M + FA) − H]^−^	C_26_H_50_O_7_NP	1.3	504.3101	1 PC ^1^ (18:2)		√			[45]
41	40.98	564.3317 [(M + FA) − H]^−^	C_26_H_50_O_7_NP	1.0	504.3317	1 PC ^1^ (16:0)		√ √			[45]

^1^ PC = PhosphatidylCholine.

Other metabolites identified in this work for the first time both in *F. vulgare* and in the Umbelliferae family were the compounds 2, 7, 12 and 18, which were identified as iridoids derivates.

Compounds 35, 36 and 54 were identified as oxylipins, in particular, the fragmentation pattern of the compounds 35 and 36 was characterized by the fragmentation of the bond in the vicinity of the hydroxyl group, producing fragment ions at *m*/*z* 211, a similar fragmentation was reported in *Abelmoschus esculentus* fruit [49], thus the compounds were identified as trihydroxy-octadecadienoic acid II, and they were for the first time reported in *F. vulgare*. 

Compounds 38 and 51 were putatively identified as gingerol and lusitanicoside, respectively; lusitanicoside was characterized on the basis of the presence of the fragment ion at *m*/*z* 133.06, due to the neutral loss of rutinoside moiety [46,50].

Compound 39 was proposed as gingerglycolipid A, which is the glycosylmonoacylglycerol of organic compounds, its fragment ion at *m*/*z* 675.3610 is like the fragmentation patterns already reported in the literature [48]. 

Compounds 40 and 41 were identified as phospholipids in the ethanolic extracts of the steam of *F. vulgare.* They were characterized by a phospholipid structure in which there is glycerol, with one fatty acylated and the nitrogenated head group corresponding to choline [45]. 

Peaks at *m*/*z* 375.1444 and 519.1858 showed the presence of two lignans, cycloolivil (43) and pinoresinol-4-*O*-glucoside (50). They were identified only in decoctions of *F. vulgare.*

**Table 2:** Metabolites identified in *F. vulgare* waste decoctions by LC-ESI/LTQOrbitrap/MS and LC-ESI/LTQOrbitrap/MS/MS analysis. If the compound is present in several parts of the fennel, the RT is referred to as the profiles of FVST, except for the compounds with * which are referred to as the profiles of FVLE.

N°	RT	[M − H]^−^	Molecular Formula	Δppm	MS/MS	Identity	FVBU	FVST	FVLS	FVLE	References
3	7.30	218.1029	C_9_H_16_O_5_N	2.8	88.0391/146.0811	pantothenic acid				√	[30]
5	8.95	343.1030	C_15_H_19_O_9_	2.0	181.0508/328.0798	methyl vanillate glucoside			√		[32]
42	12.37	555.1713	C_25_H_31_O_14_	0.9	347.1129	unknown			√		
10	12.58	353.0870	C_16_H_17_O_9_	0.8	191.0561	neochlorogenic acid		√			[9]
12	13.69	583.2018	C_27_H_35_O_14_	−0.5	375.1445/537.1970/327.1236	lucidumoside C		√	√		[37]
14	14.77	551.1761	C_26_H_31_O_13_	0.3	389.1232/491.1549/461.1456/431.1415	glehlinoside C		√			[39]
17	15.54	367.1037	C_17_H_19_O_9_	3.7	191.0566/173.0462	3-*O*-feruloylquinic acid	√				[9]
43	16.51	375.1444	C_20_H_23_O_7_	1.6	327.1228	cycloolivil			√		[44]
19	17.02	191.0349	C_10_H_7_O_4_	5.5	176.0117	6-*O*-methylesculetin			√		[40]
44	17.26	523.2174	C_26_H_35_O_11_	3.6	361.1657	secoisolariciresinol *O*-hexoside		√			[51]
21	18.36	425.1449	C_20_H_25_O_10_	1.5	263.0925	2H-1-benzopyran-6-propanoic acid, 7-(β-D-glucopyranosyloxy)-3,4-dihydro-2,2-dimethyl-4-oxo-			√		[41]
20	18.94	565.1975	C_27_H_33_O_13_	3.5	339.1249/327.1248	unknown	√	√			
45	18.98	549.1612	C_26_H_29_O_13_	1.7	387.1074	unknown			√		
24	19.92	463.0868	C_21_H_19_O_12_	−1.1	301.0359	quercitin 3-*O*-glucoside		√			[9]
25	20.77	477.0658	C_21_H_17_O_13_	−1.1	301.0352	quercitin *O*-glucuronide		√			[9]
46	20.93	389.1241	C_20_H_21_O_8_	2.5	165.0547/193.0498	resveratol 3-*O*-glucoside				√	[52]
26	21.94	605.1863	C_29_H_33_O_14_	−0.2	339.1240/327.1241	unknown		√	√		
47	22.18	563.1766	C_27_H_31_O_13_	1.2	337.1081/325.1082	7-({[(2E)-3-(3,4-dimethoxyphenyl)-2-propenoyl] oxy} methyl)-1-(glucopyranosyloxy)-1,4a,5,7a-tetrahydrocyclopenta[c]pyran-4-carboxylic acid			√		[53]
48	22.67	371.1010	C_18_H_27_O_8_	2.5	311.1516	unknown			√		
27	22.81	515.1177	C_25_H_23_O_12_	−1.4	353.0876	1,3-dicaffeoylquinic acid		√			[9]
49	23.83	199.0439	C_5_H_11_O_8_	−4.7	184.0205	unknown	√				
50	24.20	519.1858	C_26_H_31_O_11_	−0.5	357.1339	(+)-pinoresinol-4-*O*-β-D-glucoside		√			[54]
51	25.20	441.1764	C_21_H_29_O_10_	2.0	133.0647/295.1189	lusitanicoside	√			√	[50]
52	25.31	603.1704	C_29_H_31_O_14_	−0.7	337.1077	unknown		√	√		
53	25.89	271.1549	C_14_H_23_O_5_	3.5	115.0388	unknown				√	
54	27.71 *	299.1859	C_16_H_27_O_5_	2.0	183.1017/201.1124	1–14 dimetyl 2-oxotetradecanediote			√	√	[34]
55	27.91	416.2657	C_24_H_36_O_4_N_2_	−3.0	386.2552	unknown	√				
56	29.29	430.2812	C_22_H_40_O_7_N	2.9	386.2556	unknown	√				
57	29.60	273.1706	C_14_H_25_O_5_	3.7	115.0388/145.0995/255.1598	6-(2-ethyl-5-hydroxy-hexoxy)-6-oxo-hexanoic acid				√	[55]
58	30.40	491.2866	C_24_H_43_O_10_	3.2	329.2339/391.1983/313.2398	(2S)-3-(β-D-galactopyranosyloxy)-2-(hexanoyloxy)propyl nonanoate	√				[56]
59	31.82	375.0753	C_11_H_19_O_14_	−4.2	241.0020/96.9588	unknown				√	
35	32.54	327.2170	C_18_H_31_O_5_	1.3	229.1444/291.1963/211.1341/171.1027	trihydroxy-octadecadienoic acid II	√	√	√	√	[44]
36	34.44	329.2328	C_18_H_33_O_5_	1.7	229.1446/211.1343/311.2231/293.2122	trihydroxy-octadecaeoic acid II	√	√	√	√	[45]
37	36.20	363.1806	C_20_H_27_O_6_	1.1	301.1808/345.1710	cohumulinone		√	√		[46]
38	40.32	293.1765	C_17_H_25_O_4_	6.1	221.1542/236.1050	gingerol				√	[47]

### 3.2. Multivariate Data Analysis 

The chromatograms obtained by LC-ESI/LTQ-Orbitrap/MS of the ethanolic extracts and of the decoctions were subjected to chemometric analysis, and specifically to Principal Component Analysis (PCA) and Partial Least Squares-Discriminant Analysis (PLS-DA), for better understanding of the data and for evaluating the different expressions of the metabolites of the four parts of *F. vulgare* (FVBU, FVST, FVLS and FVLE).

The raw data were pre-processed with MZmine (http://mzmine.sourceforge.net/) accessed on 20 July 2020, producing two data matrices, one for the ethanolic extracts and another for decoctions with rows representing the individual samples analyzed (36 samples each data matrix: 12 biological samples in technical triplicates) and columns representing integrated and normalized peak areas (108 variables for ethanolic extracts, 120 variables for decoctions). 

**Figure 3:**
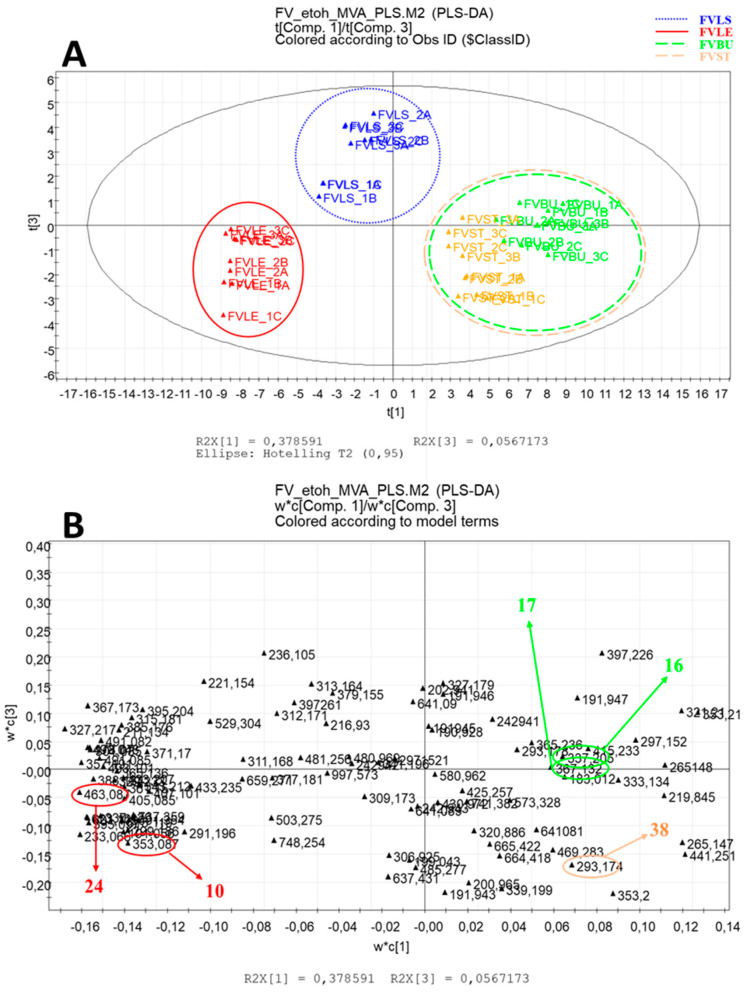
Partial Least Squares Discriminant Analysis (PLS-DA) of ethanolic extracts of *F. vulgare*. (**A**) score scatter plot; (**B**) loading plot with identifications of biomarkers.

**Figure 4:**
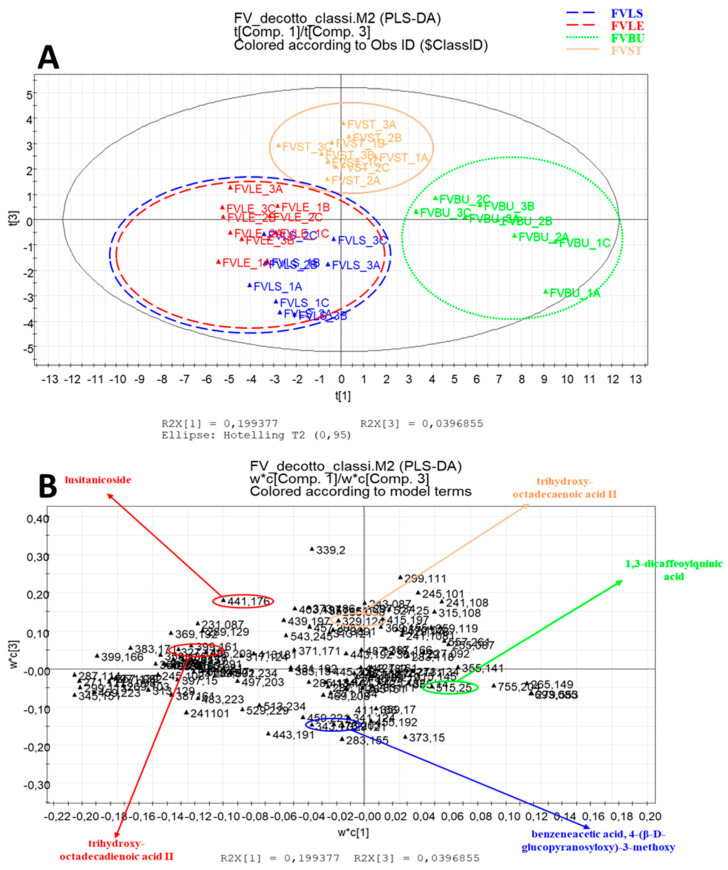
Partial Least Squares Discriminant Analysis (PLS-DA) of decoctions of *F. vulgare*. (**A**) score scatter plot; (**B**) loading plot with identifications of biomarkers.

After PCA, PLS-DA was applied; the validation of the model was obtained using a permutation test. Classification of the samples was carried out for both types of extracts, obtaining two different plots colored according to the parts of the plant under investigation (Figure 3 and Figure 4). 

The PLS-DA model representative obtained for the data acquired for ethanolic extracts showed the first component explaining 38% of the variance and the third explaining the 5% of the variance with a Q2 of 79%. The PLS-DA model representative of the decoctions showed the first component explaining 20% of the variance and the third explaining the 4% of the variance with a Q2 of 54%.

The score plot of the PLS-DA of the ethanolic extracts (Figure 3A) showed similarity in metabolic expression in the bulb and in the steam (FVBU and FVST) of *F. vulgare*, in fact, they are grouped in the lower right part of the plot. Instead, the leaf and the little steam (FVLE and FVLS) form two distinct and separate clusters in the left part of the plot.

The loading plot (Figure 3B) allowed the identification of the metabolites responsible for differentiating the samples. Increased expression of chlorogenic acid (10) and glucoside quercetin (24) was found in the leaf, while a greater expression of some quinic acid derivatives as feruloyl quinic acid (17) and coumaroyl quinic acid (16) was found in the bulb. Other metabolites expressed in the ethanolic extracts of the four parts of fennel are represented in Figure 3B.

The PLS-DA obtained for data acquired for decoctions (Figure 4A) revealed a different clustering of the samples: the bulb and stem are graphically well-differentiated from the rest of the samples, while the leaf and the little stem are more like each other and are placed in the lower left part of the plot. 

The loading plot (Figure 4B) showed that dicaffeoyl quinic acid (27) is one of the most expressed metabolites in the bulb, responsible for the sample separation from the other parts of *F. vulgare.* The leaf is rich in lusitanicoside (51) and oxylipin trihydroxy-octadecadienoic acid II (35), while the steam is high in other oxylipin trihydroxy-octadecaeoic acid II content (36). Other metabolites expressed in the decocts of the four parts of the fennel are represented in Figure 4B.

### 3.3. Antioxidant Activity and Content of Flavonoid

As a preliminary step, the antioxidant activity of the various extracts was evaluated through spectrophotometric chemical assays in which the reducing power of the samples against the radical DPPH was measured. 

**Figure 5:**
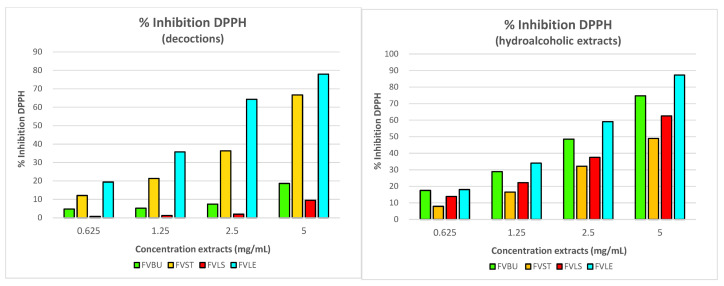
Percentage of inhibition of the radical DPPH of *F. vulgare*. The concentrations of the tested extracts are 0.625, 1.25, 2.5 and 5 mg/mL. The percent of inhibition of DPPH is the average of three experiments and the standard deviation values range from 0.01 to 0.05.

The fennel extract that showed a greater antioxidant property was one obtained from the leaf, that reached, at a concentration of 5 mg/mL, a percentage of DPPH inhibition at about 80% for decoction and about 90% for ethanol extract. While the one from the stem at the highest concentration was active in both types of extracts (50% hydroalcoholic extracts, 60% decoctions), the bulb and smaller stem showed a good percentage of inhibition only for ethanol extracts (75% FVBU and 63% FVLS) (Figure 5). 

The antioxidant activity of the various parts of the fennel was also evaluated with the TEAC assay. The higher TEAC values (Table 3) of the extracts of *F. vulgare* were shown by both types of extracts of the leaf of fennel, according to the DPPH assay. 

In addition, according to the important biological activities of flavonoids, their amount was assessed by a spectrophotometric assay, in which the flavonoids produce a yellow complex with Al^3+^, which can be revealed at a wavelength of 510 nm. The major quantity of flavonoids, expressed in rutin equivalents (RE), was found in the leaf of *F. vulgare,* with 0.206 mg/g plant extract (hydroalcoholic) and 0.263 mg/g plant extract (decoction) (Table 4). 

The antioxidant activity shown by the different parts of *F. vulgare* could be due to the metabolites characterized through the LC-ESI-FT-MS analysis, in fact, there is evidence in the literature in which the antioxidant activity of quinic acid derivatives [57] and flavonoids [58] has been studied. 

## 4. Conclusions and Discussion

The present study allowed the characterization of metabolites present in different parts of *F. vulgare* waste. The use of two different types of extraction, ultrasonically assisted hydroalcoholic extraction and decoction, showed that a higher detection of metabolites is obtained through ethanol extraction (41 metabolites) compared to decoction (38 metabolites).

Among the metabolites identified exclusively in hydroalcoholic extracts, there are: iridoids (2, 7, 13 and 18), phenylpropanoids (16 and 31), glycoside flavonoids (22, 23, 28 and 29), the glycolipid 39 and the two phospholipids 40 and 41. Furthermore, the three metabolites belonging to the class of lignans (43, 44 and 50) were identified only in the decoctions of fennel, along with the flavonoid resveratrol glucoside (46) and the oxylipin 54.

By comparing the metabolites identified in the different parts of *F. vulgare* waste, it can be observed that rutin (22) is present only in the bulb (FVBU). The representative peak of gingerol (38) in the LC-MS profile, also present in the small stems (FVLS), turns out to have greater intensity in the bulb. As for the metabolite profile of the stem (FVST), it is the only part of the fennel waste in which lipids 39, 40 and 41 were found. The flavonoids 24 and 25, present in all samples except in the FVBU, show greater intensity in the metabolite profile of the leaf (FVLE).

The interesting data that emerged from this metabolic analysis concern the presence, in each part of the *F. vulgare* waste, of the oxylipins 35 and 36, metabolites identified for the first time in fennel. The potential antitumor role of this class of metabolites has been reported in the literature [11]. In addition, there is evidence that many plant species belonging to the family Apiaceae, known for their potential cytotoxic, anti-inflammatory and anticancer properties, have been reported to contain metabolites belonging to this class of compounds, suggesting that such biological properties may be due not only to phenolic compounds but also to oxylipins [59].

Chemometric analyses enabled a better interpretation of the metabolic analysis data and made clearer the different metabolic expressions of the four classes of samples analyzed (FVBU, FVST, FVLS and FVLE). Although Principal Component Analysis and Partial Least Square Analysis of the samples obtained through hydroalcoholic extraction were not identical to those obtained for decoctions, it is evident from both score plots that the bulb and stem show more similar metabolic expression than the leaf and the small stem.

Both the assay to evaluate the content of flavonoids and those to evaluate the antioxidant activity of the different parts of fennel suggest that the leaf is the most active part. Based on those data, the loading plots of multivariate statistical analysis propose which metabolites are most expressed in FVLE samples (10, 24, 35, 51), and which could be the cause of the best biological activities of the *F. vulgare* leaf, making it the best source of bioactive compounds and therefore the most promising part to be valorized with the aim to produce functional or nutraceutical products.

The metabolomics approach here described allowed the characterization of the parts of fennel not used in the food industry and destined to become vegetable waste. These parts of *F. vulgare* are rich in bioactive compounds potentially useful for producing functional and nutraceutical products. It would be interesting to isolate these metabolites to evaluate the biological activities of individual compounds, with the aim to produce functional products enriched with bioactive compounds.

## Figures and Tables

**Table 3:** Antioxidant activity of extracts of *F. vulgare* evaluated by Trolox Equivalent Antioxidant Capacity (TEAC).

*F. vulgare* Extracts	TEAC [mg/mL ± SD ^a^] ^b^
FVBU Hydroalcoholic	0.334 ± 0.007
FVBU Decoctions	0.393 ± 0.003
FVST Hydroalcoholic	0.347 ± 0.004
FVST Decoctions	0.426 ± 0.014
FVLS Hydroalcoholic	0.375 ± 0.006
FVLS Decoctions	0.407 ± 0.011
FVLE Hydroalcoholic	0.823 ± 0.008
FVLE decoctions	0.570 ± 0.009

^a^ SD: Standard Deviation of three independent experiments; ^b^ Determined by TEAC assay.

**Table 4:** Total amount of plant flavonoids in *F. vulgare* extracts.

*F. vulgare* Extracts	Total Flavonoids [mg/g Plant Extract (in RE) ± SD ^a^] ^b^
FVLE hydroalcoholic	0.206 ± 0.006
FVLE decoctions	0.263 ± 0.005

^a^ SD: Standard Deviation of three independent experiments; ^b^ Determined by Allumine Chloride assay.

## Data Availability

Not applicable.
